# Corrosion Behaviour of Weld Metal of Ultra-High-Strength Steel Weldments in a Sodium Chloride Aqueous Solution

**DOI:** 10.3390/ma17184534

**Published:** 2024-09-15

**Authors:** Mariana Ilieva, Danail Gospodinov, Nikolay Ferdinandov, Rossen Radev

**Affiliations:** Department of Materials Science and Technology, University of Ruse “Angel Kanchev”, 8 Studentska St., 7017 Ruse, Bulgaria; dgospodinov@uni-ruse.bg (D.G.); nferdinandov@uni-ruse.bg (N.F.); rradev@uni-ruse.bg (R.R.)

**Keywords:** ultra-high-strength steel, manual arc welding, weld metal, corrosion, microstructure, potentiodynamic scan

## Abstract

As high-strength and ultra-high-strength steels are widely used in all kinds of modern welded constructions, a lot of research is carried out to investigate the mechanical properties of the weldments of these steels, but there is little information on such important characteristics as their corrosion behaviour. This research focuses on the corrosion behaviour of the weld metal of the weldments of S906QL and S700MC steels. The weld metal was tested electrochemically in a 3.5% NaCl aqueous solution via a potentiodynamic scan to determine the corrosion rate and its dependence on the welding gap. No influence of the welding gap on the corrosion rate was found, but the experimental results suggested that the corrosion rate depended on the chemical composition of the filler material and the microstructure of the weld metal.

## 1. Introduction

The need for energy savings and environmental preservation has led to the development of high-strength steels. Structural steels with a high yield point are known as high-strength steels (HSSs), and when the yield point of the steels is above 700 MPa, they are called ultra-high-strength steels (UHSSs) [1]. Both HSS and UHSS have various practical uses, ranging from construction to automotive and spacecraft production. In the modern world, HSS and UHSS play a key role in global warming, as these steels offer high strength combined with reduced vehicle weight and different structures, thus lowering harmful emissions. For instance, lighter trucks made of high-strength steel can carry more goods while emitting the same amount of CO_2_ per kilometre [2]. As HSS and UHSS offer the same or increased strength at reduced weight compared to conventional steels, their use allows more energy-efficient and cost-efficient designs for vehicles, wind turbines, transformers, motors, and other types of construction. This leads to a reduction in energy consumption throughout the lifespan of the equipment and construction. Therefore, HSS and UHSS enhance structural performance and also support environmental preservation by reducing emissions and promoting sustainable practices.

High-strength steels obtain their mechanical properties after careful steel composition design and heat treatment or thermo-mechanical treatment. An example of heat-treated ultra-high-strength steel is S960QL. S960QL has a high yield strength (up to 960 MPa depending on the thickness) due to the combination of the proper choice of alloying elements and heat treatment—quenching and tempering. S700MC is another example of ultra-high-strength steel. The high yield strength of S700MC (700 MPa) is achieved by a combination of microalloying and thermo-mechanical treatment, i.e., rolling at elevated temperatures and then accelerated cooling.

Both grades possess good weldability; therefore, S960QL and S700MC weldments are widely used in different welded structures. Numerous studies have been conducted on the structure and mechanical properties of S960QL and S700MC weldments [3,4,5,6,7,8,9,10,11,12,13,14,15]. Unfortunately, information on the corrosion behaviour of the weldments of both steels is hard to find in the specialised literature. It is well known that different zones in the weldments and base metal demonstrate different corrosion rates, and this leads to the formation of corrosion galvanic elements. Nevertheless, a more detailed exploration work on the corrosion behaviour of weldments is needed to assess the lifespan of welded structures and the most probable spots for corrosion to occur to prevent the loss of mechanical stability and structural integrity.

The present paper focuses on the determination of the corrosion rates of the weld metal (fusion zone) of the weldments of S960QL and S700MC in the most abundant electrolyte on our planet, i.e., the sodium chloride water solution, using accelerated electrochemical testing. As the use of a standardised weld gap is not suitable in various applications, the weldments tested here were made with different welding gaps, and the influence of the welding gaps on the corrosion of weld metal was experimentally found.

## 2. Materials and Methods

The tested specimens were weldments of S960QL and S700MC. The detailed welding procedures are described in [16,17]. Both steels were manufactured by Voestalpine Stahl GmbH (Linz, Austria). S960QL came as a plate with a thickness of 6 mm, and S700MC came as hot-rolled sheets with a thickness of 8 mm. The chemical composition of the steels, according to the manufacturer, is given in Table 1.

The welded parts had dimensions of 500 × 150 × 6 mm for S960QL and 500 × 150 × 8 mm for S700MC (length, width, thickness), following ISO 15614-1:2017 [18]. As the welding method, submerged arc welding was used, with direct current and reverse polarity DC (+). Four different welding gaps of 0 mm, 4 mm, 6 mm, and 8 mm were used as described in [16,17]. Except for the welding gap root, which had to be investigated for its impact on the weldments, the joint preparation followed ISO 9692-2:2001 [19]. Without a root face, a mechanical bevel was performed at an angle of 30° (for a total of 60°). As a filler material, a wire of AWS ER120S-G for S960QL was used, and for S700MC, a wire of AWS ER 100S-G was used, manufactured by ESAB (North Bethesda, MD, USA). In both cases, the diameter of the filler material was 1.2 mm, and the flux was S A AB 1 56 AC H5 (ISO 14174:2019 [20]). The chemical composition of the filler materials, according to the manufacturer, is shown in Table 2, and the welding parameters are shown in Table 3.

Test specimens of the weldments were cut off after welding to examine the macrostructure and microstructure and to perform corrosion tests. The macrostructure was examined in two directions—parallel to the welding direction and normal to the welding direction. Prior to etching for macrostructural analysis, the specimens were wet ground with 240, 320, 400, 500, 600, 800, and 1000 SiC grit. For macrostructure evaluation, a saturated FeCl_3_ (ITW Reagents, Barcelona, Spain) water solution was used at room temperature for 15 s. Metallographic examination was performed on the microsections normal to the welding direction after wet grinding and mechanical polishing with Al_2_O_3_ 0.5 µm (Leco, St. Joseph, MI, USA) and final polishing with Al_2_O_3_ 0.03 µm (Leco, St. Joseph, MI, USA). A 3% HNO_3_ (ITW Reagents, Barcelona, Spain) alcohol solution was used for etching the microsections for 5 s. An optical Epitip 2 (Carl Zeiss Jena, Jena, Germany) microscope equipped with a digital camera was used to observe the microstructure and to take microphotographs of it. In the Results Section, only a few representative microphotographs are given.

**Figure 1:**
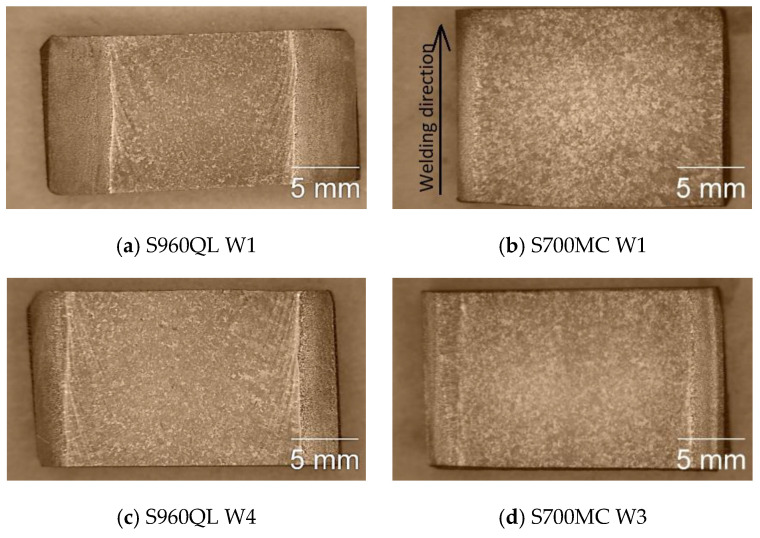
Macrostructure of the weldments, longitudinal section.

The corrosion rate was evaluated using accelerated electrochemical testing. Electrochemical tests were carried out on the weld metal surface, parallel to the welding direction, as shown in Figure 1. The tested surfaces were ground and polished for metallographic examination and cleaned with acetone. As an aggressive environment, a neutral 3.5% NaCl (ITW Reagents, Barcelona, Spain) water solution was chosen as this is the average sodium chloride concentration in the world’s oceans [21]. Prior to the electrochemical test, every specimen was allowed to stabilise in the electrolyte for 50 min. At the end of this stabilisation period, the steady state potential E_ss_ was measured against a saturated calomel electrode SCE. The potential of the SCE at the test room temperature (the temperature of the solution was 19 °C) was +245 mV against the standard hydrogen electrode SHE, and all the values of the potentials presented here are calculated according to this value against the SHE. Following this was linear polarisation using a standard three-electrode cell with the tested sample as a working electrode, the SCE as a reference electrode, and a Pt-counter electrode. The potential was controlled using a Radelkis OH 105 (Radelkis, Budapest, Hungary) potentiostat equipped with a National Instruments USB-6008 controller (National Instruments, Austin, TX, USA) connected to a computer. The area of the tested surfaces was 0.5 cm^2^, and the rate of potential increase was 1 mV/s, starting from −650 mV up to −250 mV. eL-ChemViewer software, version 3.3 [22] was used for Tafel analysis for corrosion current density i_corr_ and corrosion potential E_corr_ determination. Using the obtained i_corr_ values, the corrosion penetration rate CR was calculated, as described in [23]. To assure experimental reliability, three specimens of every welding mode, as well as of the two base metals, were tested. Here, the averaged values are presented.

## 3. Results

The present work aims to characterise the weld metal. First, to evaluate the corrosion behaviour of the weld metal, macrostructural analysis of the weld metal of the weldments was performed. Figure 1 presents the macrostructure of the surfaces of some of the tested weldments. The weld metal (WM) on the longitudinal sections of both steels looks similar, except for the visible isotherms on the S960QL weld metal—curved lines, showing how the pool moved during welding. Near the WM boundaries, curved dendrites are observed for both steels’ weldments, and approaching the centre line of the weldments, the shape of the WM changes to an equiaxed one.

**Figure 2:**
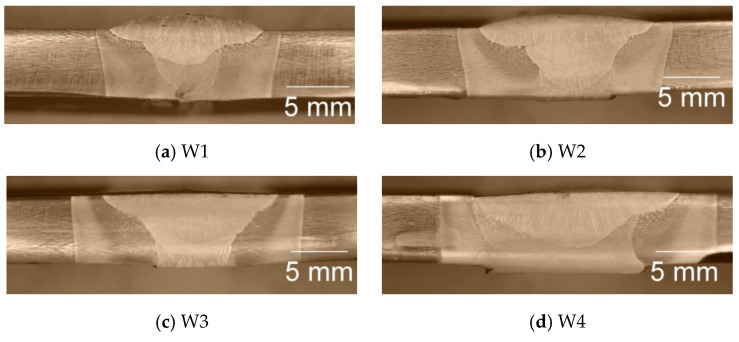
Macrostructure of weldments of S960QL, cross-section.

**Figure 3:**
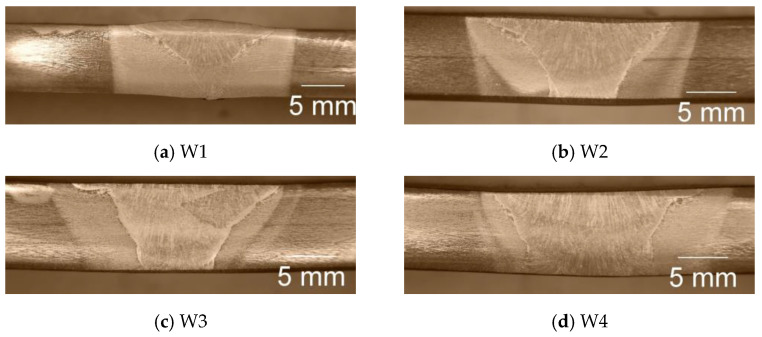
Macrostructure of weldments of S700MC, cross-section.

To find the faults and depth of fusion, cross-sections of the samples of both steels were observed, as shown in Figure 2 and Figure 3.

For all the welding modes, faults are not observed, but the weldments show full penetration. With the increase in the weld gap dimension, the width of the sum of the different welding zones increases too, as was expected. It is interesting to note that the coarse-grained heat-affected zone on the weldments of S700MC is narrower than the observed one on the weldments of S960QL. The weld metal of all the weldments demonstrates a typical dendritic (cast) structure with grains elongated towards the weld root near the weld centre line and toward the heat-affected zone near the ends of the WM. Increasing the distance between the welded parts leads to an increase in the weld’s asymmetry. A detailed description of the macrostructure of the weldments studied here is given in [16,17].

**Figure 4:**
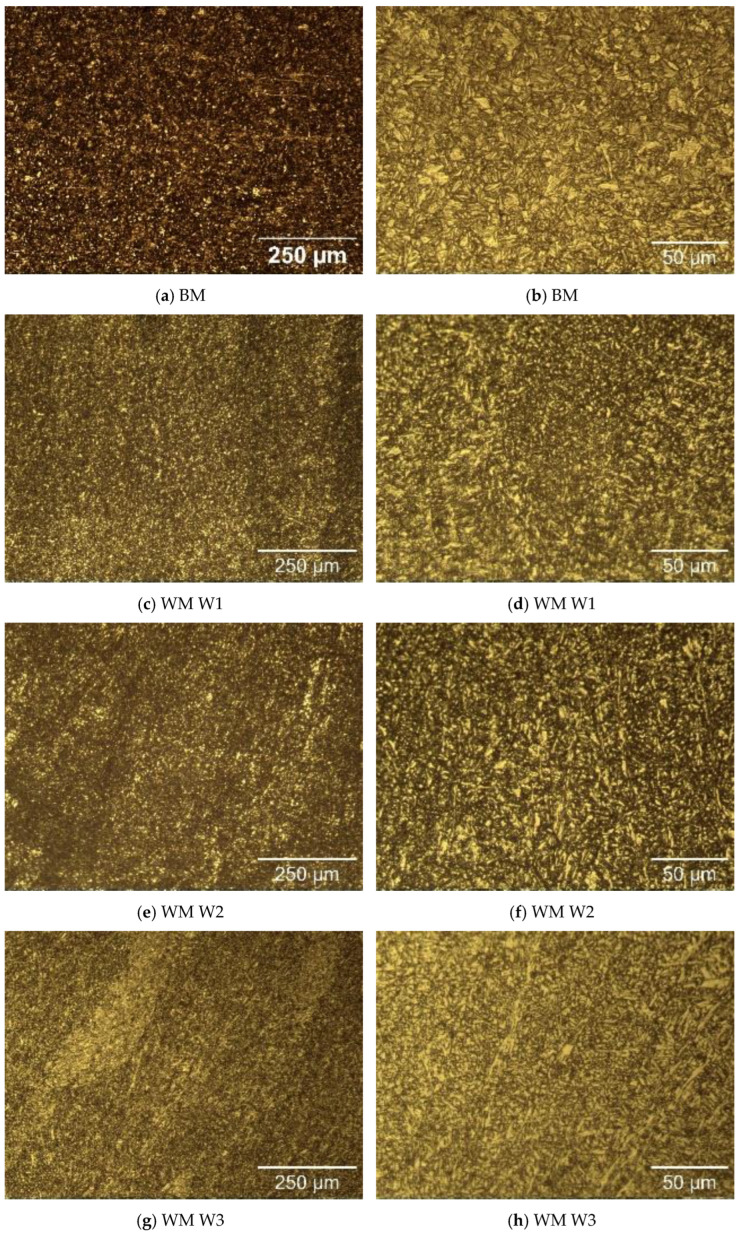

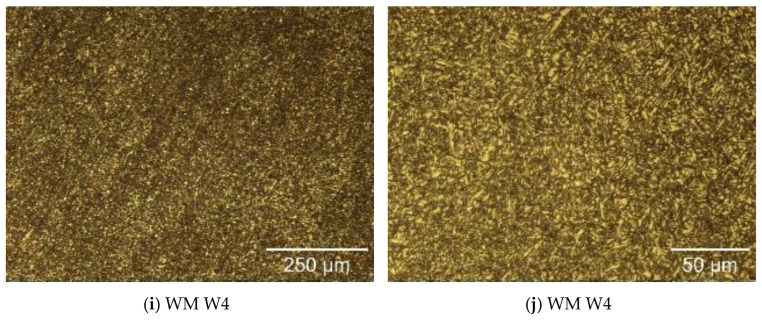
Microstructure of the base metal (BM) and weld metal (WM) of the weldments of S960QL at two different magnifications.

**Figure 5:**
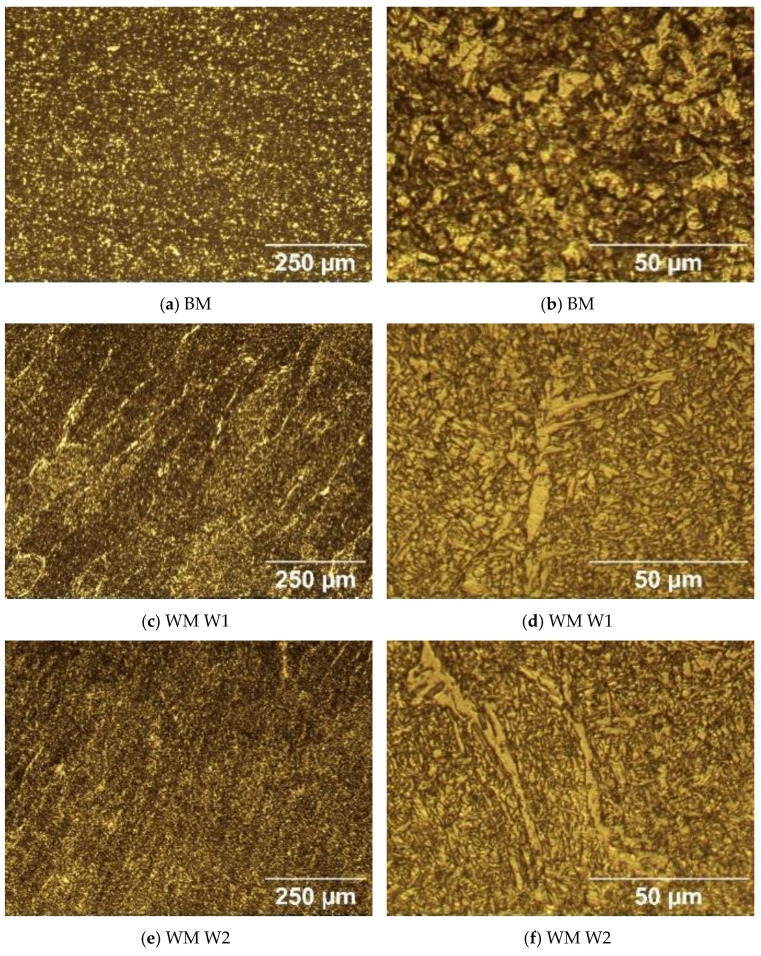

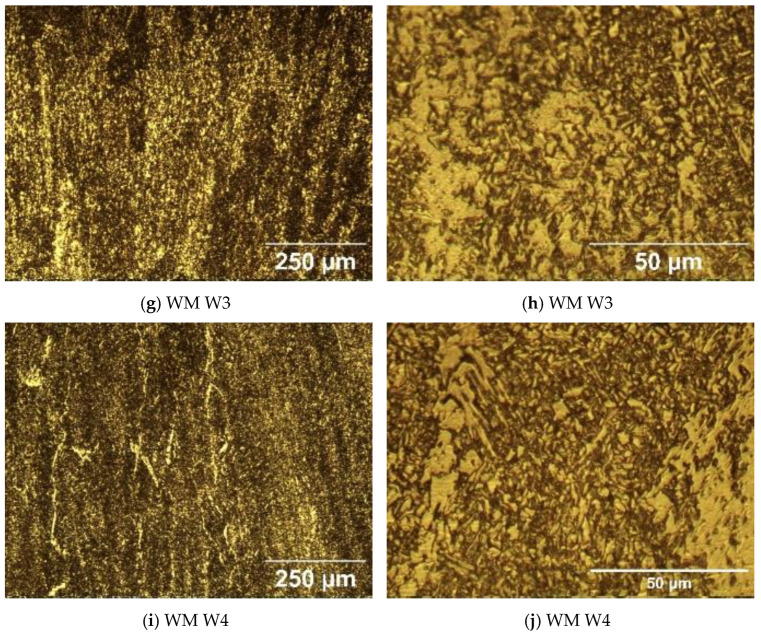
Microstructure of the base metal (BM) and weld metal (WM) of the weldments of S700MC at two different magnifications.

The microstructure of the specimens of S960QL is presented in Figure 4, and the microstructure of the specimens made of S700MC is given in Figure 5. The S960QL steel demonstrates a homogeneous-looking microstructure, consisting of tempered martensite and bainite—Figure 4a,b. The microstructure of the S700MC steel is composed of ferrite and bainite [17] and shows signs of the thermo-mechanical treatment the steel was subjected to by the manufacturer—Figure 5a,b. The microstructure of the weld metals of all the weldments demonstrates a cast structure with a white constituent as elongated grains—Figure 4 and Figure 5. This white constituent is ferrite and is larger in terms of share and dimensions in the WM of the S700MC than in the WM of the S960QL weldments, as is visible in Figure 4 and Figure 5.

The tested surfaces are shown in Figure 1. As the opening of the specimens’ holder for corrosion testing was 8 mm and the specimens were centred in the holder, the results presented here only give information about the corrosion behaviour of the weld metal near the centre line of the weldments.

**Figure 6:**
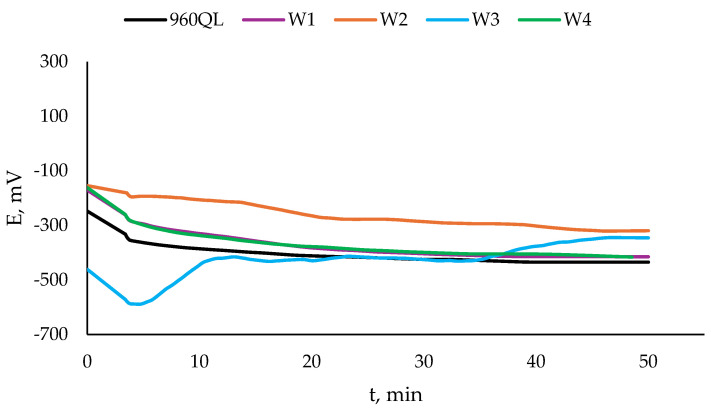
Open circuit potential of S960QL and weld metal of weldments of S960QL in a 3.5% NaCl water solution at room temperature.

**Figure 7:**
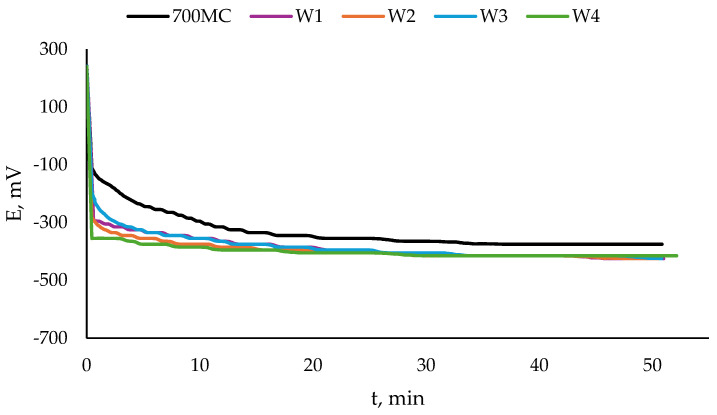
Open circuit potential of S700MC and weld metal of weldments of S700MC in a 3.5% NaCl water solution at room temperature.

The open circuit potential OCP and its change in time are shown in Figure 6 for the S960QL specimens and in Figure 7 for the S700MC specimens. The values of steady state potential E_ss_ are summarised in Table 3. While S960QL demonstrates more negative OCP and E_ss_ than the weld metal of the weldments of S960QL, S700MC displays a more noble potential than its weldments.

**Figure 8:**
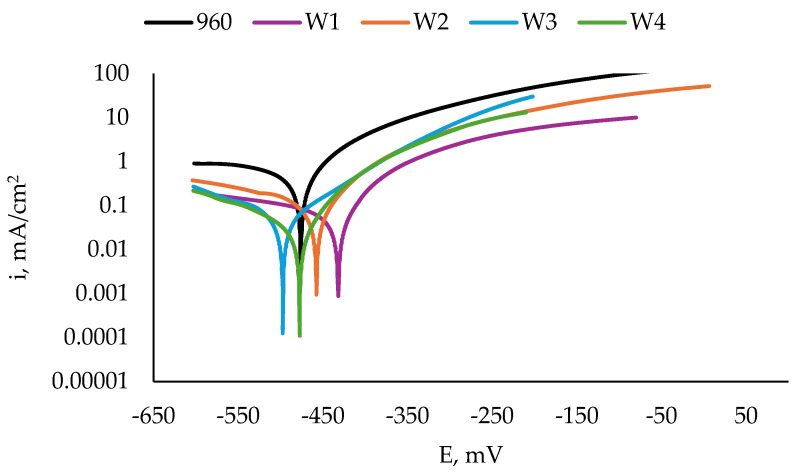
Polarisation curves of S960QL and weld metal of weldments of S960QL in a 3.5% NaCl water solution at room temperature.

**Figure 9:**
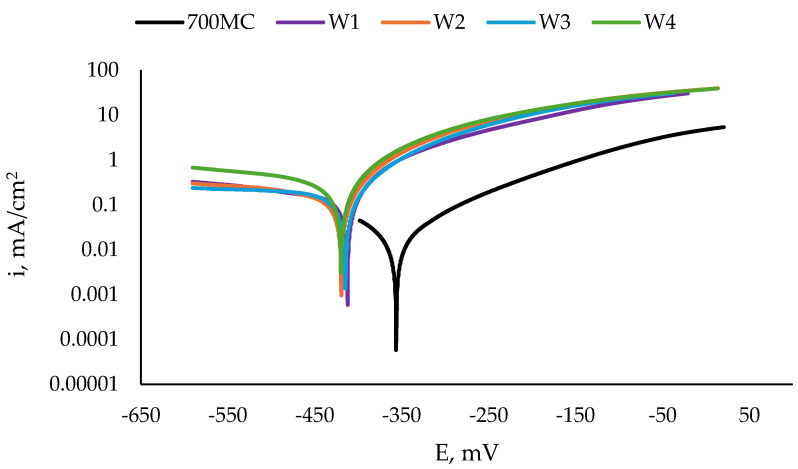
Polarisation curves of S700MC and weld metal of weldments of S700MC in a 3.5% NaCl water solution at room temperature.

Figure 8 shows the results from the potentiodynamic scan of the specimens of S960QL, and Figure 9 shows those of the S700MC specimens. Corrosion potential E_corr_, corrosion current density i_corr_, and calculated corrosion penetration rate CR are presented in Table 3. It is seen in Figure 8 that the weld gap does not categorically influence the corrosion potential of WM on S960QL—the weld metal of the weldments made by welding modes W1 and W2 has slightly more positive values of E_corr_, while the corrosion potential values of the weld metal of the weldments obtained by welding modes W3 and W4 are shifted in a negative direction. Nevertheless, regarding corrosion current density, the corrosion rates of the weld metal of all the weldments of S960QL are lower by one order of magnitude than those of the base metal. A different picture is observed for the specimens made of S700MC—the corrosion potential of the weld metal of all the specimens of S700MC is shifted towards more negative values compared to the base metal, and the corrosion current density and corrosion rate outweigh the corresponding values for the base metal by one order of magnitude. No local corrosion damage was observed after electrochemical testing, but uniform surface dissolution was seen.

## 4. Discussion

The corrosion behaviour of the weld metal of the weldments studied here should be governed by their structure and the specific corrosion medium. The difference in chemical composition between the base metal and weld metal must also be taken into account when discussing practical implications.

*The role of the medium*. The formation of thick stable protective passive layers for both steels in the used neutral solution or in air is not possible; so, the tested surfaces are unprotected from the action of aggressive ions. As the sodium chloride concentration in the solution is low, oxygen solubility is at its highest levels, and there is enough dissolved oxygen to reach the cathodic areas on the specimens’ surfaces and for the corrosion process to take place without an effective diffusion—a barrier film to prevent the anodic dissolution of the specimens [24]. Thus, one can expect that the main factors controlling the dissolution of the specimens are their structure and chemical composition.

*The role of structure and chemical composition*. As described in [25], the addition of Ni to a medium-carbon ultra-high-strength steel leads to a decrease in the corrosion rate. This effect is profound up to 0.5 wt.% Ni, and the next increase up to 1 wt.% in Ni content only slightly decreases the corrosion rate, but the open circuit potential increases consistently with Ni addition to an acid medium. The beneficial effect of Ni on the corrosion rate of medium-carbon steel in a neutral NaCl aqueous solution is attributed to the formation of a thin, rich Ni layer under the outer layer of iron oxides [25].

S960QL and S700MC both have a bainite constituent, but the S700MC base metal also reveals an equilibrium constituent—ferrite grains—while the microstructure of the S960QL base metal is an entirely non-equilibrium microstructure: tempered martensite and bainite. Despite the higher concentration of Ni and Cu in S960QL, this non-equilibrium microstructure and the micro-stresses connected to it led to the higher corrosion rate of S9600QL compared to the S700MC base metal.

The weld metal was characterised by a dendritic structure, but due to the directional crystallisation, opposite to the heat output, the grains on all tested surfaces of the weld metal showed an almost equiaxed shape. The microstructure of the weld metal of the S700MC specimens was characterised by a larger amount of idiomorphic ferrite. According to [26], idiomorphic ferrite in weld metal forms when non-metallic inclusions, mostly oxides, serving as nucleation sites for the heterogeneous nucleation of ferrite, are present in the weld metal. This indicates that oxygen in a larger amount entered the weld pool during welding. The presence of idiomorphic ferrite was more pronounced for the weldments of S700MC obtained by welding modes W3 and W4, but it did not affect the corrosion behaviour of the weld metal, as is visible in Figure 7 and Figure 9 and Table 4.

The Ni concentration in S960QL was 0.039 wt.%, and in the filler metal, it was 2.22 wt.%, i.e., more than 56 times higher. The real cooling rates during welding did not allow a significant diffusion of Ni atoms toward the base metal, thus leaving the nickel concentration as the highest in the weld metal. During the non-equilibrium phase transformation following crystallisation, most of Ni atoms remained locked in martensite and retained austenite, and some of them remained in the newly formed ferrite. As the weldments were not subjected to heat treatment, the structural stresses in the weld metal were higher than those in the base metal. Thus, the corrosion behaviour of the weld metal of the weldments of S960QL was a result of two opposing phenomena: (1) A local enrichment of weld metal in Ni, decreasing the corrosion rate and (2) a high level of structural stresses, increasing the corrosion rate. As is visible in Figure 6 and Figure 8 and Table 4, none of these phenomena prevails when only OCP, E_ss_, and E_corr_ are considered, but obviously, the above-mentioned richness in the Ni layer led to some protection of the weld metal, and the corrosion rate decreased.

The weld metal of the weldments of S700MC showed worsening corrosion behaviour compared to the base metal. Apparently, the chemical composition of the filler material did not work to keep the corrosion potential and steady state potential unchanged. The electrochemical reaction on the weld metal’s surface occurred with cathodic control as the steady state and corrosion potentials of the weld metal were close to the standard electrode potential of pure iron. Nevertheless, both the anodic and cathodic current densities of the weld metal of the S700MC specimens were higher than those of the base metal. Thus, the non-equilibrium microstructure of the weld metal was the factor which controlled the corrosion behaviour of the weld metal of the S700MC samples, and it resulted in greater the corrosion rates of the weld metal.

For the weld metal of all the weldments of both steels, the welding gap did not affect the corrosion behaviour.

As the steady state and corrosion potentials of the weld metal of all the weldments of S700MC were shifted in a negative direction, the appearance of a corrosion galvanic cell could be expected with the weld metal acting as an anode and the base metal acting as a cathode. Since the potential difference between the base metal and weld metal is not so pronounced (40 to 50 mV), the resulting current should not have high values. Nevertheless, the base metal surpasses the weld metal in area, i.e., the base metal represents a cathode consuming a considerable number of electrons. Thus, in real working environments that promote corrosion processes, a high corrosion rate and dissolution of the weld metal is to be expected.

The appearance of a corrosion galvanic cell is also expected for the specimens of S960QL, but in this case, the role of the anode and cathode could not be categorically specified. Despite this, preventive measures should be considered when employing weldments of S960QL and S700MC, and the practical one is corrosion protection painting.

## 5. Conclusions

The results presented in this work focus on the corrosion behaviour of weld metal. The weldments consist of more zones than a weld metal and to completely characterise them future research must be conducted; however, the experiments presented here add to the scarce information about the corrosion behaviour of the weldments of high-strength steels.

The practical conclusion about the influence of the width of the welding gap on the corrosion behaviour of weld metal is that when designing welding modes no consideration with respect to corrosion is needed, as the width of the welding gap does not affect the corrosion behaviour of the weld metal of the weldments of S960QL and S700MC in 3.5% NaCl aqueous solution.

The corrosion behaviour of the weld metal depends on its microstructure and the chemical composition of the filler materials, and neither of these two factors always prevails. Thus, the filler material for the welding of S960QL leads to a decrease in the corrosion rate of the weld metal despite its non-equilibrium microstructure, but the corrosion behaviour of the weld metal of the weldments of S700MC is governed by its non-equilibrium microstructure, and the filler material does not affect it in 3.5% NaCl aqueous solution.

For practical purposes, corrosion prevention of the weldments of S960QL and S700MC with paints is recommended.

## Figures and Tables

**Table 1:** Chemical composition of S960QL and S700MC steels.

**Chemical Composition of S960QL in Weight %**															
**C**	**Si**	**Mn**	**P**	**S**	**Al**	**B**	**Cr**	**Cu**	**Mo**	**N**	**Nb**	**Ni**	**Ti**	**V**	**Zr**
0.175	0.249	1.075	0.009	0.001	0.084	0.0023	0.620	0.018	0.608	0.0039	0.029	0.039	0.003	0.002	0.001
**Chemical Composition of S700MC in Weight %**															
**C**	**Si**	**Mn**	**P**	**S**	**Al**	**B**	**Cr**	**Cu**	**Mo**	**N**	**Nb**	**Ni**	**Ti**	**V**	**Zr**
0.065	0.049	1.830	0.006	0.0006	0.051	0.0002	0.025	0.009	0.002	-	0.049	0.009	0.123	0.008	-

**Table 2:** Chemical composition of AWS ER120S-G and AWS ER100S-G, according to manufacturer.

**Chemical Composition of AWS ER120S-G in Weight %**					
C	Si	Mn	Ni	Cr	Mo
0.081	0.80	1.75	2.22	0.41	0.53
**Chemical Composition of AWS ER100S-G in Weight %**					
C	Si	Mn	Ni	Cr	Mo
0.12	0.71	1.38	0.53	0.58	0.20

**Table 3:** Welding modes and parameters at different welding gaps.

Welding Mode (W)	Welding Gap, mm	Pass №	Current I, A	Voltage U, V	Welding Rate, mm/min	Scheme of Welding Sequences
W1	0	1	240	31	280	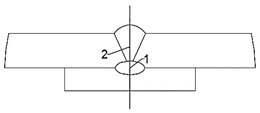
		2	270	31	150	
W2	4	1	240	31	250	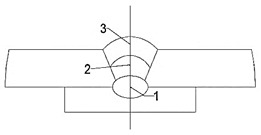
		2	240	31	180	
		3	270	32	140	
W3	6	1	240	31	250	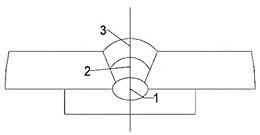
		2	240	31	180	
		3	270	32.5	120	
W4	8	1	240	31	250	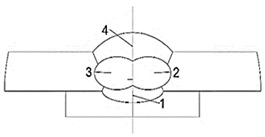
		2	240	31	180	
		3	240	31	180	
		4	270	32.5	100	

**Table 4:** Some electrochemical characteristics of the tested specimens.

Welding Mode	E_ss_, mV		E_corr_, mV		i_corr_, mA/cm^2^		CR, mm/yr		Microstructure	
	S960QL	S700MC	S960QL	S700MC	S960QL	S700MC	S960QL	S700MC	S960QL	S700MC
Base metal	−435	−375	−476	−357	3.47 × 10^−1^	1.40 × 10^−2^	4.04	0.16	M + B	F + B
W1	−415	−425	−440	−414	6.80 × 10^−2^	1.30 × 10^−1^	0.79	1.50	M + A_ret._ + F_al._	M + A_ret._ + F_id._
W2	−320	−425	−458	−421	4.46 × 10^−2^	1.40 × 10^−1^	0.52	1.58		
W3	−346	−424	−494	−417	5.56 × 10^−2^	1.60 × 10^−1^	0.65	1.85		
W4	−416	−415	−481	−428	4.43 × 10^−2^	3.30 × 10^−1^	0.52	3.81		

E_ss_—steady state potential; E_corr_—corrosion potential; i_corr_—corrosion current density; CR—corrosion penetration rate; M—martensite; A_ret._—retained austenite; B—bainite; F_al._—allotriomorphic ferrite; F_id._—idiomorphic ferrite.

## Data Availability

The original contributions presented in the study are included in the article, further inquiries can be directed to the corresponding author.
